# Understanding lipotoxicity in NAFLD pathogenesis: is CD36 a key driver?

**DOI:** 10.1038/s41419-020-03003-w

**Published:** 2020-09-25

**Authors:** Patricia Rada, Águeda González-Rodríguez, Carmelo García-Monzón, Ángela M. Valverde

**Affiliations:** 1 Instituto de Investigaciones Biomédicas Alberto Sols (CSIC/UAM), 28029 Madrid, Spain; 2 Centro de Investigación Biomédica en Red de Diabetes y Enfermedades Metabólicas Asociadas (CIBERDEM, ISCIII), 28029 Madrid, Spain; 3 Unidad de Investigación, Hospital Universitario Santa Cristina, Instituto de Investigación Sanitaria del Hospital Universitario de La Princesa, 28009 Madrid, Spain; 4 Centro de Investigación Biomédica en Red de Enfermedades Hepáticas y Digestivas (CIBEREHD), 28029 Madrid, Spain

**Keywords:** Prognostic markers, Metabolic syndrome, Obesity, Non-alcoholic fatty liver disease, Non-alcoholic steatohepatitis

## Abstract

Non-alcoholic fatty liver disease (NAFLD) is the most common cause of chronic liver disease worldwide. NAFLD stages range from simple steatosis (NAFL) to non-alcoholic steatohepatitis (NASH) which can progress to cirrhosis and hepatocellular carcinoma. One of the crucial events clearly involved in NAFLD progression is the lipotoxicity resulting from an excessive fatty acid (FFA) influx to hepatocytes. Hepatic lipotoxicity occurs when the capacity of the hepatocyte to manage and export FFAs as triglycerides (TGs) is overwhelmed. This review provides succinct insights into the molecular mechanisms responsible for lipotoxicity in NAFLD, including ER and oxidative stress, autophagy, lipoapotosis and inflammation. In addition, we highlight the role of CD36/FAT fatty acid translocase in NAFLD pathogenesis. Up-to-date, it is well known that CD36 increases FFA uptake and, in the liver, it drives hepatosteatosis onset and might contribute to its progression to NASH. Clinical studies have reinforced the significance of CD36 by showing increased content in the liver of NAFLD patients. Interestingly, circulating levels of a soluble form of CD36 (sCD36) are abnormally elevated in NAFLD patients and positively correlate with the histological grade of hepatic steatosis. In fact, the induction of CD36 translocation to the plasma membrane of the hepatocytes may be a determining factor in the physiopathology of hepatic steatosis in NAFLD patients. Given all these data, targeting the fatty acid translocase CD36 or some of its functional regulators may be a promising therapeutic approach for the prevention and treatment of NAFLD.

## Facts

- Hepatic lipotoxicity, due to massive FFAs flux from peripheral tissues or enhanced de novo lipogenesis, has been associated to NAFLD severity and comprises a variety of processes such as ER and oxidative stress, autophagy, apoptosis and inflammation.
- CD36 fatty acid translocase drives hepatosteatosis onset, therefore contributing to NAFLD progression.
- Growing evidences suggest that CD36 could be a potential biomarker for NAFLD diagnosis and patient’s stratification.

## Open questions

- What are the key signalling pathways involved in liver-specific regulation of fatty acid translocase CD36 expression and function?
- Which are the major drivers linking hepatic lipotoxicity to increased CD36 function within the liver?

## Introduction

Nowadays, non-alcoholic fatty liver disease (NAFLD) is the commonest cause of chronic liver disease worldwide^1,2^. The global prevalence of NAFLD is thought to be constantly increasing, being currently estimated ~25%^2^. Moreover, the presence of coexisting risk factors such as obesity and type 2 diabetes (T2D) increases its prevalence up to ~55%^1^. Since most patients with NAFLD present some of the well-known features of metabolic syndrome (MS) such as central or visceral obesity, glucose intolerance, systemic hypertension and dyslipidemia, NAFLD is now considered the hepatic manifestation of obesity and MS.

NAFLD represents a spectrum of well-defined stages encompassing simple fatty liver (NAFL), non-alcoholic steatohepatitis (NASH) and fibrosis. NAFL is mostly a benign non-progressive clinical entity, whereas NASH, a more severe condition, likely progresses to cirrhosis causing liver failure and the need for liver transplantation and, ultimately, hepatocellular carcinoma (HCC). Based on the increased NAFLD frequency in the last decade, this disease will become the most common indication for liver transplantation between 2020 and 2025^3^. Histological analysis of liver sections revealed that NAFL is characterised by macrovesicular steatosis with or without nonspecific inflammation, whereas NASH includes hepatic steatosis associated with evidences of liver cell injury, ballooning degeneration of hepatocytes, inflammation and presence of Mallory-Denk bodies (MDBs) and apoptotic bodies. Because of the disease complexity, scoring systems were developed to help pathologists in assessing the severity of NAFLD^4^.

### Lipotoxicity in NAFLD: a broad spectrum of molecular mechanisms

Different theories have emerged in order to understand the underlying mechanism for the development and progression of NAFLD. According to the traditional “two-hit” hypothesis, hepatic accumulation of lipids secondary to sedentary lifestyle, high-fat diet (HFD), obesity and insulin resistance, acts as the first hit, sensitising the liver to a second hit that activates inflammatory cascades and fibrogenesis^5^. However, a more accurate explanation of NAFLD pathogenesis contemplates that several molecular and metabolic changes take place synergistically in its development and progression. This fact gave rise to the “multiple hit” hypothesis that considers multiple insults acting together to induce NAFLD^6^. Such hits include insulin resistance and lipotoxicity, among others.

Hepatic lipotoxicity occurs when the liver capacity to use, store and export FFAs as triglycerides (TGs) is overwhelmed by a massive FFA flux from the periphery, mainly the adipose tissue, or by increased hepatic de novo lipogenesis, both hallmarks of insulin resistance and NAFLD. Certainly, FFAs levels correlate with disease severity^7–9^. Saturated FFAs are more hepatotoxic than unsaturated species. In vitro studies demonstrated that monounsaturated palmitoleate (POA) (C16:1) and oleate (OA) (C18:1) are less toxic than saturated FFAs such as palmitate (PA) (C16:0) or stearate (SA) (C18:0)^9–11^, probably due to the ability of unsaturated FFAs to be esterified into neutral TGs in a more efficient manner^12^. Also, unsaturated FFAs counteract PA-induced toxicity in hepatocytes^9,13,14^.

**Fig. 1:**
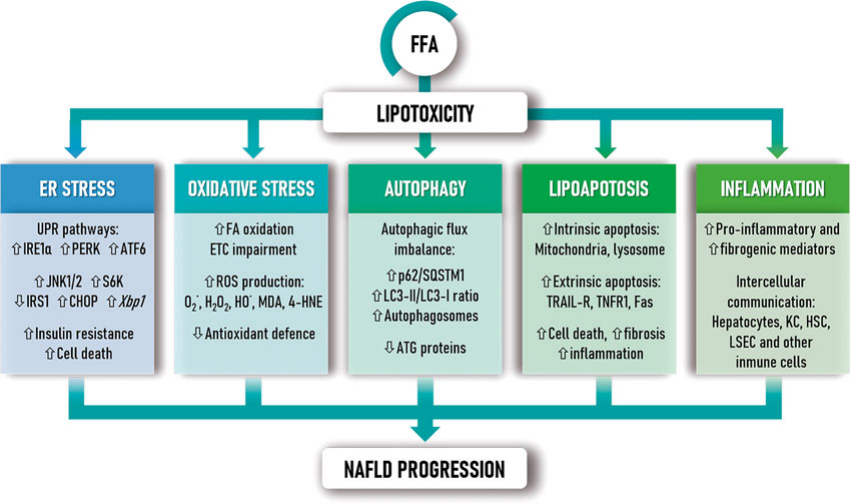
Lipotoxic effects mediated by FFAs contribute to NAFLD progression. FFAs-induced lipotoxicity promotes ER and oxidative stress, insulin resistance and impairs autophagy. As a consequence, FFAs activate apoptotic cascades thus promoting tissue damage and inflammation. Altogether, these molecular events contribute to NAFLD progression.

Recent advances in the underlying cellular processes behind lipotoxicity will be reviewed herein to understand their impact in NASH development and progression (Fig. 1). In addition, a particular emphasis will be laid on discussing the role of CD36/FAT (CD36, fatty acid translocase) in the evolution of NAFLD.

### Endoplasmic reticulum (ER) stress and lipotoxicity

**Fig. 2:**
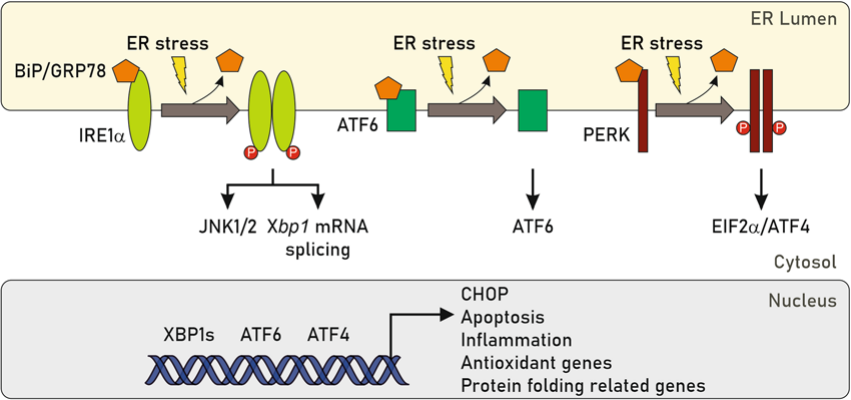
Endoplasmic reticulum (ER) stress-induced unfolded protein response (UPR) signalling. ER stress signalling involves three main protein sensors, PRKR-like endoplasmic reticulum kinase (PERK), inositol-requiring enzyme-1α (IRE1α) and activating transcription factor-6 (ATF6). These proteins remain inactive while are bound to the intraluminal chaperone glucose-regulated protein 78 (GRP78), also named Binding Protein (BiP). In response to ER stress, these mediators become activated and released, thereby triggering molecular cascades that activate the unfolded protein response (UPR). Overall, activation of each sensor promotes ATF4, XBP1s and ATF6 translocation to the nucleus to induce the expression of their relevant target genes associated with apoptosis, inflammation, antioxidant response and protein folding mechanisms, among others, to restore ER homeostasis.

Hepatic ER stress occurs upon excessive accumulation of unfolded and misfolded proteins in the ER or when ER calcium is depleted. Three main sensors trigger ER signalling cascades aimed to restore ER homeostasis^15^ (Fig. 2). A large number of studies have established that exposure to saturated FFAs promotes ER stress mainly in hepatocytes^9,16,17^. Particularly, Wang and colleagues reported that livers from rats fed a diet enriched in saturated fat (HSAT) showed spliced *Xbp1* (Unspliced X-box-binding Protein 1) mRNA, increased GRP78 (glucose-regulated protein 78 or Binding Protein, BiP) and the apoptotic transcription factor CHOP (CCAAT-enhancer-binding protein homologous protein) compared to rats fed a diet enriched in polyunsaturated fat^18^. Interestingly, similar findings were shown in primary hepatocytes isolated from those mice. Comparable results were reported in rats fed a high sucrose (HS) diet, suggesting that an increase in hepatic saturated FFAs derived from either peripheral lipids (HSAT diet) or accelerated de novo lipogenesis (HS diet) promotes ER stress and liver injury. Subsequent studies demonstrated that direct exposure to saturated FFAs, such as PA or SA, disrupts ER homeostasis and induces apoptosis in hepatocarcinoma cell lines^16,17^ as well as primary hepatocytes from human or mouse origin^9^.

The impact of saturated FFAs in ER homeostasis was also examined in mice with global deletion of the gene encoding stearoyl-CoA desaturase-1 (SCD1), a critical enzyme that catalyzes conversion of saturated to monounsaturated FFAs. *Scd1*^−/−^ mice exhibited a marked ER stress manifested by enhanced *Xbp1* splicing and CHOP levels in the liver^19^. Conversely, increased lysophosphatidylcholine acyltransferase 3 (LPCAT3) activity counteracts saturated FFAs-induced ER stress in hepatocytes and mouse liver^20^.

**Fig. 3:**
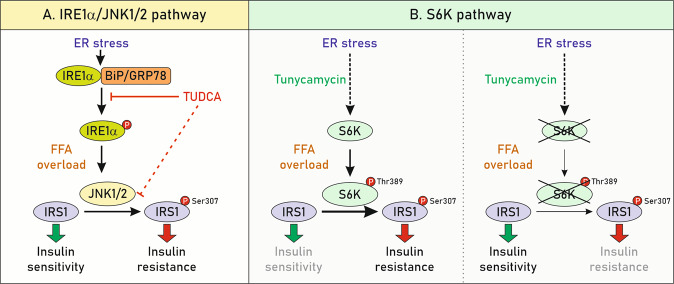
FFAs-mediated ER stress signalling contributes to hepatic insulin resistance during NAFLD. **a** JNK1/2, activated by IRE1α, promotes inhibitory serine phosphorylation of the insulin receptor substrate 1 (IRS1)^139^, which interrupts insulin-dependent signalling. The key role of IRE1α–JNK1/2 was evidenced by the fact that the chaperone TUDCA, an ER stress inhibitor, blocked the activation of JNK1/2 and IRS1 serine phosphorylation, thereby preserving the integrity of the insulin signalling cascade. **b** S6K1 is phosphorylated at Thr389 in response to PA^9,140^, a mechanism described earlier to mediate IRS1 phosphorylation at Ser307^141^. In line with these findings, S6K1 deficiency in hepatocytes blocked the effects of tunicamycin, a classical ER stressor, and also ameliorated PA-induced insulin resistance, suggesting that targeting S6K1 is an attractive strategy against ER-mediated lipotoxicity and insulin resistance^9^.

Although there is no a conclusive mechanism explaining how saturated FFAs induce ER stress, increasing evidence points that Jun N-terminal kinase 1/2 (JNK1/2) and ribosomal protein S6 kinase 1 (S6K) pathways are undoubtedly involved in this process. In fact, JNK1/2 activation has been reported in NASH patients as well as in murine models of NASH^21^. In addition, in vitro experiments revealed that saturated, but not unsaturated FFAs, induce JNK1/2 activity^10^. Notably, ER stress signalling induced by FFAs also modulates hepatic insulin resistance associated to NAFLD progression^22^ (Fig. 3).

### Oxidative stress and lipotoxicity

Oxidative stress plays a fundamental role in the initiation and progression of NAFLD. Clinical studies showed elevated oxidative stress and lipid peroxidation in patients with NAFLD^23,24^. Mitochondria are the most significant source of reactive oxygen species (ROS) in NAFLD, mainly due to increased fatty acid oxidation (FAO) upon FFAs overload^25^. Briefly, FFAs are converted into fatty acyl-CoAs in the cytoplasm and further transferred to the mitochondria via carnitine palmitoyltransferase 1 (CPT1). Once fatty acyl-CoAs reach the mitochondrial matrix, they are decomposed through FAO to form acetyl-CoA that is metabolised through the tricarboxylic acid (TCA) cycle. Overactivation of FAO and TCA cycle induces an electron overflow through the electron transport chain (ETC). Whereas in healthy conditions electrons are shuttling through cytochrome c oxidase and combining with oxygen and protons to form H_2_O, impairment of the ETC promotes leakage of electrons and subsequent activation of molecular oxygen to form superoxide (O_2_^•^) and hydrogen peroxide (H_2_O_2_)^25^. Alternatively, peroxisomes oxidise long-chain FFAs more efficiently than mitochondria, thus increasing the cell capacity to metabolise these lipid species. However, peroxisomal β-oxidation produces H_2_O_2_, which is rapidly transformed into the highly reactive hydroxyl radical (HO^•^)^26^.

One of the key consequences of uncontrolled oxidative stress is a direct damage to lipids^27^. ROS can initiate lipid peroxidation by targeting polyunsaturated fatty acids resulting in the formation of highly reactive aldehyde products such as 4-hydroxy-2-nonenal (4-HNE) and malondialdehyde (MDA). Importantly, these reactive compounds have longer half-lives than free radicals and can diffuse into the extracellular space and boost tissue damage. A large number of studies have reported that both MDA and 4-HNE are increased in experimental animal models of NASH (methionine-choline-deficient diet -MCD-, high fat-high sucrose diet -HFHSD-) as well as in NASH patients in comparison to patients with NAFL^23,28^. Moreover, cytochrome P450, lipoxygenase and cyclooxygenase, well-characterised pro-oxidant systems, in combination with free radical products have been implicated in the early stages of NAFLD^29,30^. On the other hand, a reduction in the activity of antioxidant enzymes such as catalase, glutathione peroxidase, glutathione S-transferase, superoxide dismutase, as wells as ROS scavengers (ascorbic acid, glutathione (GSH), α-tocopherol, ubiquinone, thioredoxin, and bilirubin) is a feature of livers from NASH patients^28,30,31^. Therefore, a tight control of ROS levels by antioxidants might be important to restore redox homeostasis. Since nuclear factor erythroid-2-related factor 2 (NRF2) enhances the expression of most of these antioxidant enzymes and down-regulates the expression of genes involved in fatty acid synthesis^32^, several studies have reported that targeting NRF2 is crucial in the protection against NASH^33^. In line with these findings, mice lacking *Nfe2l2* (encodingNRF2) fed a MCD^34,35^ or HFD^36^ recapitulate many NASH features, suggesting a key role of NRF2 in preventing NASH progression, not only due to the activation of antioxidant genes, but also by modulating fatty acid metabolism in hepatocytes.

### Role of autophagy in lipotoxicity

Autophagy is a catabolic process that degrades intracellular organelles via lysosomal pathway to maintain energy homeostasis during periods of nutrient deprivation or to eliminate undesirable cellular components. In the last decade many studies have suggested that dysregulation of autophagy is implicated in NAFLD pathogenesis^37^. Notorious data have been reported on the regulation of autophagic processes by unsaturated and saturated FFAs in primary hepatocytes and hepatocarcinoma cell lines^38,39^. In this regard, a previous study from our laboratory^38^ demonstrated that short-term treatment of Huh7 human hepatic cells with PA (8 h) activated the autophagic flux, an effect reflected by reduced mTORC1 and S6K1 phosphorylation, decreased p62/SQSTM1 levels and increased LC3-II/LC3-I ratio in agreement with the guidelines for monitoring autophagy-related assays published by Klionsky et al.^40^. As no apoptotic cells were detected at this time-period, these findings suggest that rapid activation of autophagic flux is a protective response against cell death. In contrast, prolonged exposure of Huh7 cells to PA (24 h) induced accumulation of autophagosomes and cell death. These data clearly indicate that prolonged PA treatment leads to a switch from activation to inhibition of the autophagic flux. These findings were also documented in HepG2 cells loaded with PA for 24 h, where decreased autophagic flux was associated with activation of caspase-3 and apoptosis^39^. Moreover, this study demonstrated that OA promotes the formation of TG-enriched lipid droplets, induces autophagy, and has little effect on apoptosis. Based on these evidences, the differences in toxicity between saturated and unsaturated FFAs might be due, at least in part, to their differential capacity to modulate the autophagic machinery.

Growing evidences support that impairment of autophagic flux also occurs in preclinical models of NAFLD such as mice fed a HFD, MCD or HF-high-cholesterol diet (HFHCD)^38,41^. For instance, in livers from mice fed a HFD or MCD, persistent activation of ER stress-mediated signalling, assessed by elevations in phospho-PERK, GRP78/BiP or CHOP levels, paralleled the blockade of autophagic flux measured by increases in p62/SQSTM1, LC3-II/LC3-I ratio and accumulation of autophagosomes compared with mice fed a standard diet. Remarkably, inhibition of ER stress restored the autophagic flux in hepatocytes cultured in MCD medium, suggesting a reciprocal regulation between these two processes. Altogether, these results indicate that all the machinery that connects ER stress with the blockade of autophagic flux is already activated in mice with hepatic steatosis. Studies in animal models targeting autophagy-related genes have evidenced the contribution of this process to NAFLD development. Mice deficient in *Sqstm1* (p62/SQSTM1; named *p62* KO) develop mature-onset obesity together with insulin and leptin resistance when fed a standard diet^42^. However, another study in the same animal model revealed that NAFLD progression to NASH and tumorigenesis occurred only by additional deletion of the gene encoding NRF2^43^. Moreover, liver-specific knockdown of *Atg7* or *Atg14* markedly increased hepatic TG and cholesterol content indicating that defects in autophagy promote hepatic steatosis^44–46^. By contrast, restoration of *Atg7* or *Atg14* in the liver successfully reversed hepatic steatosis in *ob/ob* mice and mice challenged with a HFD, respectively. This phenomenon was associated with restoration of autophagy and decreased ER stress^45,46^.

In addition to the preclinical studies, we and others found accumulation of p62/SQSTM1 together with an increase in LC3-II/LC3-I ratio in livers from patients with NAFL and NASH^38,41^. More importantly, p62/SQSTM1 levels were significantly higher in NASH patients compared with those with NAFL, suggesting that this effect is related to disease progression in humans. Besides, NASH patients displayed more elevated ER stress markers, such as GRP78/BiP and CHOP, reinforcing the notion that enhanced ER stress within liver cells may be relevant in the progression from NAFL to NASH in humans. In view of the pro-survival function of autophagy in protecting hepatic cells against lipotoxicity, potential therapeutic strategies aimed to restore the autophagic flux might contribute to prevent or attenuate the progression of NAFLD.

### Lipoapoptosis as an end-point in lipotoxicity associated with NAFLD

The process of apoptosis or programmed cell death normally occurs during development and aging as a homeostatic mechanism to preserve healthy cell populations in tissues. However, pathological conditions can trigger apoptosis in order to eliminate damaged cells^47^. In the context of NAFLD, this process is referred as lipoapoptosis because it is believed to be secondary to a massive lipid deposition in hepatic cells^48^. In this regard, the magnitude of lipoapoptosis associates with the degree of inflammation and the stage of fibrosis, suggesting that it might be a cellular mechanism distinguishing NASH from benign steatosis^48,49^.

Failure of the hepatocyte to manage the excess FFAs by packaging into TGs is associated with increased hepatocyte lipoapoptosis. In this sense, SCD1 plays a pivotal role by converting saturated to monounsaturated FFAs. While SCD1 overexpression increases TG synthesis and protects against lipoapoptosis, genetic deletion of *Scd1* (encoding SCD1) aggravates hepatocyte apoptosis and liver damage^50^. Similarly, knockdown of diacylglycerol acyltransferase 2 (DGAT2), a key enzyme in the esterification of FFAs to TGs, potentiates liver injury in MCD-fed mice^51^.

**Fig. 4:**
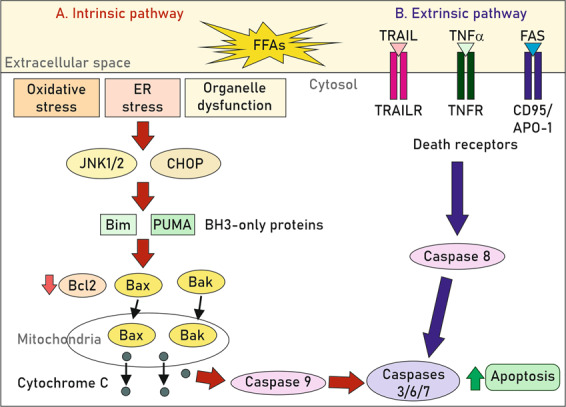
FFAs activate apoptosis via intrinsic or extrinsic pathways. Whereas the intrinsic apoptotic mechanism is initiated by intracellular stimuli such as oxidative stress, ER stress or organelle dysfunction, the extrinsic pathway is activated in response to external stimuli, namely by binding of death ligands, such as TNF-related apoptosis-inducing ligand (TRAIL), TNFα or Fas (CD95/APO-1), to their respective death receptors (DR) in the cell surface. In hepatocytes, the apoptotic signals from DR are not robust enough to trigger the effector caspase cascade, so the intrinsic pathway is also activated to boost the apoptotic response^142,143^. The induction of the intrinsic pathway involves a decrease of anti-apoptotic proteins such as Bcl2 and the translocation of pro-apoptotic members (Bax, Bak) to the mitochondria triggering cytochrome c release and other apoptosis-inducing factors to the cytosol, thereby activating procaspase-9 and downstream apoptotic effectors^143^.

Saturated FFAs can activate apoptosis via intrinsic or extrinsic pathways that are not mutually exclusive^52^ (Fig. 4). To date, it is well-characterised that FFA-induced ER and oxidative stress activate numerous signalling pathways, including CHOP- and JNK-dependent upregulation of pro-apoptotic BH3-only proteins, mainly Bim and PUMA, leading to Bax activation that directly links to the intrinsic apoptotic pathway^48^. In addition, death receptor (DR) activation by FFAs has been extensively investigated. The impact of the extrinsic apoptotic signalling pathways mediated by DR in NASH/NAFLD experimental models, as well as in cultured hepatocytes, is summarised in Table 1.

**Table 1:** Death receptor activation by FFAs in cultured hepatocytes, mouse NAFLD models and humans.

*TRAIL/TRAILR*		Ref.
Human	*TNFRSF10B* mRNA elevated in livers from patients with NASH.	^144^
Human hepatocytes	TRAILR2 (D5), but not TRAILR1 (D4) is activated after PA exposure promoting apoptosis via caspase 8.	^145^
	Exacerbated ER stress increases protein levels and activation of TRAILR2.	^146^
	*TNFRSF10B* (TRAILR2) knockdown attenuates FFAs-induced apoptosis.	^147^
Mouse	Increased *Tnfrsf10b* (encoding TRAILR), the mouse single ortholog for both human *TNFRSF10A* and *TNFRSF10B* (encoding TRAILR1 and TRAILR2, respectively) in mice fed a MCD diet.	^148^
	*Tnfrsf10b* null mice displays reduced steatosis, hepatocyte apoptosis, macrophage-associated inflammation and fibrosis after a high-fructose, high-fat, high-cholesterol diet (HFHFHCD).	^149^
*TNFα/TNFR1*		
Human	TNFα and its receptor 1 (TNFR1), are both upregulated in NASH patients.	^150,151^
Human hepatocytes	Treatment of HepG2 cells with a mixture of OA and PA (2:1) resulted in lysosomal permeabilization associated with Bax translocation and release of cathepsin B which, in turn, evoked the induction of *Tnfa* mRNA and promoted a modest rate of apoptosis. However, it is not clear whether both cathepsin B-dependent effects are related to each other.	^152^
Mouse	*Tnfrsf1a* (encoding TNFR1)-deficient mice fed a HS diet did not exhibit hepatic steatosis despite the fact that they became obese.	^152^
	*Tnfrsf1a* gain of function mutation aggravates NAFLD features.	^153^
	HFD-fed *Tnfrsf1a* knockout mice displayed an exacerbated inflammatory response, insulin resistance and liver steatosis, suggesting a cross-talk between the TNFR1 and lipid accumulation in the liver that promotes an accelerated progression from NAFL towards a more severe phenotypes of NAFLD.	^154^
*Fas ligand/Fas (CD95, APO-1)*		
Human	Fas expression is enhanced in NASH patients.	^8,155^
Human hepatocytes	HepG2 hepatocytes treated with a mixture of OA/PA showed upregulated Fas expression and increased sensitivity to Fas-mediated apoptosis.	^156^
Mouse	Liver-specific Fas overexpression compromises FAO and mitochondrial respiration, promoting lipid accumulation and insulin resistance.	^157^
	Pharmacological or genetic Fas depletion in the liver protects mice from hepatic steatosis and insulin resistance in NASH mice models.	^155,157^
Mouse and Human	In normal liver, the hepatocyte growth factor receptor Met binds to Fas, preventing its activation, whereas in both human and experimental NAFLD Fas sequestration by Met is abrogated, favouring the formation of Fas-Fas ligand complexes, and eventually inducing apoptosis.	^158^

Taking into consideration that key components of the apopotic pathways, such as caspase and DR activation, are also implicated in both pro-inflammatory and fibrogenic responses in non-parenchymal liver cells (NPCs)^49,53^, the above-mentioned studies and many others not discussed in this review support that inhibition of pro-apoptotic signalling may serve as a therapeutic strategy against NASH. To this end, different anti-apoptotic agents have been developed and are currently in clinical trials (reviewed in refs. ^54,55^).

### Lipotoxicity-induced inflammation in the interactome between liver cells populations

**Fig. 5:**
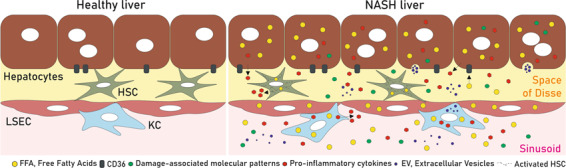
Intercellular communication between liver cells is altered in NASH livers. In healthy liver, different hepatic cell types such as hepatocytes, KCs, HSCs and LSECs, communicate and regulate each other by secreting signalling mediators. Upon NAFLD condition, after FFAs overload, damaged cells release higher amounts of pro-inflammatory cytokines, damage-associated patterns (DAMPs), extracellular vesicles (EVs) and other molecules that can activate HSCs and KCs and promote LSECs to lose their fenestrations, likely contributing in a coordinated manner to the progression of NASH to more severe stages of NAFLD.

Damaged hepatocytes during lipotoxicity release cytokines, chemokines, extracellular vesicles (EVs) and other intracellular molecules that can activate liver NPCs including Kupffer cells (KCs), hepatic stellate cells (HSCs) and liver sinusoidal endothelial cells (LSECs) as well as the recruitment of other immune cells populations^56,57^. Consequently, sustained tissue inflammation and excessive scarring results in advanced fibrosis and, ultimately, in cirrhosis. Hence, the intrahepatic interactome between different cell types via secreted factors links lipotoxicity with inflammation and fibrosis, thus accelerating the progression of NAFL to NASH^57,58^ (Fig. 5).

Hepatic macrophages are populations of KCs and macrophages that arise from infiltrated bone marrow-derived monocytes. During NASH, KCs acquire a pro-inflammatory phenotype and secrete multiple cytokines and chemokines, which in turn triggers the recruitment of other immune cells, including monocytes, boosting liver inflammation^59^. The direct effect of FFAs in macrophages has been reported by our group and others^60,61^. RAW 264.7 macrophages, as well as primary KCs treated with PA, switched towards a pro-inflammatory polarisation stage reflected by elevated TNFα, IL6, IL1β and inducible nitric oxide synthase (iNOS). However, this effect was not detected in macrophages exposed to OA that presented an opposite anti-inflammatory phenotype^60^. Likewise, KCs from HFD-fed mice showed increases in the expression of the pro-inflammatory cytokines TNF-α and IFN-γ^61^. The in vivo relevance of macrophages was assessed by depletion of KCs or inhibition of macrophage influx into the liver in murine models of NASH^62^. CC-chemokine receptor 2 (*Ccr2*)^−/−^, Toll-like receptor (*Tlr*)4^−/−^, *Tlr*9^−/−^, and *MyD88*^−/−^ mice had reduced steatosis, inflammatory cells infiltration and fibrosis after a choline-deficient diet (CDD). Accordingly, KCs depletion also delayed disease progression in several NASH mice models^62,63^.

In addition to the direct effect of FFAs in the immune populations of the liver, growing evidences indicate that EVs released by lipotoxic hepatocytes (Hep-EVs) activate macrophages in the context of NAFLD. In this regard, Kakazu and colleagues demonstrated that PA-mediated ER stress stimulates EVs release from hepatocytes that promoted macrophage chemotaxis because they contained sphingosine 1-phosphate (S1P), a ceramide metabolite that activates its receptor present in the membrane of macrophages^64^. Also, another study reported that pro-apoptotic/lipotoxic signalling triggered by mixed lineage kinase 3 (MLK3) induces the release of Hep-EVs enriched in potent C-X-C motif chemokine ligand 10 (CXCL10) leading to monocyte-derived macrophages chemotaxis to the liver and may activate KCs during NASH progression^65^.

Besides macrophages/KCs, the liver recruits a broad spectrum of NPCs such as neutrophils and T lymphocytes cells. Although the immune signature of NAFLD is not the main topic of this review, it is noteworthy to mention a recent study in humans showing a direct correlation between circulating and hepatic cytotoxic CD8^+^ T lymphocytes and histological hallmarks of NASH^66^ that might serve as potential biomarker for NAFLD patient’s stratification.

HSCs, also termed perisinusoidal cells, Ito cells, lipocytes or fat-storing cells, are NPCs residing in the space of Disse and constitute ~8% of the total cells in a healthy liver. In NAFLD, HSCs become activated and rapidly lose their lipid droplets and produce extracellular matrix components (i.e. α-smooth muscle actin, desmin, type I and III collagens and fibronectin) and pro-fibrogenic cytokines (TGFβ). In primary human HSCs, exposure to either PA or OA induced cellular stress by different mechanisms^67^. Whereas PA induced a transient expression of the ER stress marker CHOP, OA decreased CHOP expression and increased Thioredoxin Interacting Protein TXNIP, an inflammasome activator^67^. This raises the possibility that upon exposure to FFAs, HSCs not only promote a fibrogenic response, but also provoke an inflammatory response by releasing cytokines and enhancing cytotoxic damage in the surrounding cells. In agreement, CCL5 and CCL20, potent chemokines originated from both hepatocytes and activated HSC, are increased in serum from individuals with NASH^68,69^.

LSECs, the most abundant NPCs in the liver, play an essential role in the regulation of the transport of macromolecules between the blood and liver parenchyma including lipids and lipoproteins. LSECs have both pro- and anti-inflammatory functions in the liver^70,71^. However, their contribution to NAFLD progression is still under investigation. Recently, it has been reported that culture of LSECs with OA/PA (1:1) up-regulates lipid metabolic pathways and down-regulates pro-inflammatory chemokines^72^. Accordingly, LSECs from HFD-fed mice displayed significantly lower expression of CCL2, CXCL10 and CXCL16. Since NASH is characterised by infiltration of pro-inflammatory cells in the context of increased hepatic FFAs uptake, the authors hypothesised that LSECs may provide a compensatory mechanism whereby they downregulate chemokines and help in preventing disease progression. Supporting this hypothesis, it was shown that LSECs inhibit HSCs activation and attenuate fibrosis development^73^. However, in the presence of an uncontrolled hepatocyte lipotoxicity and injury, LSECs might lose their regulatory functions and rapidly become dysfunctional, therefore contributing to disease.

In summary, NPCs showed differential responses to lipid overload that likely contribute to the coordinated progression of NASH to more severe stages of NAFLD. These studies and many others not mentioned in this review involve liver-resident cells other than hepatocytes as new players in the complex interactome underlying NAFLD pathogenesis.

### Functions of CD36 in liver cells: not only fatty acid uptake is important

As previously mentioned, an excess of circulating FFAs is particularly harmful for the liver since they induce toxicity and, even, apoptosis in hepatocytes. FFA cellular uptake is based on a simple passive diffusion process, so it depends on intracellular and extracellular concentrations; likewise, it can also be regulated by transport proteins in response to mechanical and hormonal stimulus. Several transport proteins have been implicated in cellular FFA uptake including the family of Fatty Acid Transport Proteins (FATPs), the plasma membrane Fatty Acid Binding Protein (FABPpm), caveolin 1 and the fatty acid translocase CD36^74^.

CD36 was initially identified in platelets as an 88 kDa membrane glycoprotein, and subsequently on the surface of a wide variety of cells: macrophages, adipocytes, myocytes, enterocytes and hepatocytes^75^. CD36 is a multifunctional signalling molecule with several known ligands such as thrombospondin 1, long-chain FFAs, the native lipoproteins HDL, LDL and VLDL, and modified lipoproteins, including oxidised LDL (oxLDL)^76^. Therefore, CD36 can function in a wide range of processes including apoptosis, angiogenesis, phagocytosis, thrombosis, inflammation, atherosclerosis and FFA uptake.

The role of CD36 in lipid metabolism was evidenced by unravelling its functions as a macrophage receptor for oxidised LDL and as an adipocyte receptor/transporter for long-chain FFAs^77^. It is now known that CD36 plays an important role in facilitating intracellular FFA uptake and trafficking, as well as in esterification of FFAs into TGs in cardiac and skeletal muscle cells^78^. Importantly, overexpression of CD36 confers tissues an increased capacity to FFAs and lipoprotein influx and/or utilisation.

There are increasing evidences showing that CD36 not only acts as a FFA transporter, but also regulates FFAs oxidation, lipid synthesis, VLDL secretion, inflammation and autophagy in liver cells. To highlight, while oxidation of exogenous FFAs has been shown to be reduced in *Cd36*-deficient mice and humans due to impaired FFA uptake^79^, Samovsky et al.^80^ convincingly demonstrated that *Cd36*-knockout mice showed enhanced FAO of endogenous TG stores in myocytes by an AMPK-dependent mechanism, indicating that CD36 regulates AMPK activation linking FFA uptake to FAO. Regarding lipid synthesis, a reduced rate of hepatic de novo lipogenesis (DNL) has been observed in *Cd36*-deficient mice while FFA uptake was largely unaffected^81^. These intriguing findings may be due to the impaired glucose homeostasis seen in *Cd36-*deficient mice, but the relationship between CD36 and hepatic DNL requires further experimental exploration. Interestingly, a potential role of CD36 in regulating hepatic VLDL secretion was proposed by Nassir et al.^82^ as they observed that *Cd36* deletion in *ob/ob* mice exacerbated liver steatosis mainly due to reduction in hepatic secretion of VLDL-TGs, ApoB48 and ApoB100. In addition, CD36 appears to play a role in regulating autophagy in hepatocytes since it was recently reported that its deficiency in mice increased autophagy, while reconstitution of *Cd36* expression in *Cd36*-deficient mice reduced autophagy^83^. This study also demonstrated for the first time that *Cd36* knockdown in hepatocytes increased autophagy by an AMPK-dependent mechanism which contributed to counteract lipid accumulation, indicating that CD36 is a negative regulator of autophagy^83^. Further investigations in animal models are needed to fully elucidate the significance of CD36 regulating autophagy in vivo. So far, there is a robust scientific evidence that CD36 exerts pleiotropic actions regulating lipid homeostasis in many cell types, but little is known about the relationship between CD36 and lipotoxicity. Shedding light on this matter, Zhao et al.^84^ noticed that palmitoylation of CD36 facilitated fatty acid uptake, impaired FAO, activated the pro-inflammatory JNK/NF-κB pathway and enhanced lipid accumulation in hepatocytes, whereas inhibition of CD36 palmitoylation protected mice from developing NASH and inhibited the JNK signalling in hepatocytes. Whether CD36 palmitoylation could be a pathogenic driver linking CD36 to hepatic lipotoxicity deserves to be fully elucidated.

### Regulation of CD36 expression and function in liver cells

Under physiological conditions, the expression of CD36 in the hepatocyte is very weak, but its expression is highly inducible in the liver by lipid overload or activation of nuclear receptors^85^. On one side, CD36 expression increased concomitantly with the hepatic TG content in different animal models of hepatic steatosis^86–89^. It has also been described that experimental reversion of NAFL was accompanied by a significant reduction of hepatic CD36 levels in mice^90–92^. In fact, prolonged exposure to FFAs increased CD36 protein content in primary hepatocytes and hepatocarcinoma cell lines^93–95^. On the other hand, as the *Cd36* gene contains a peroxisome-proliferator response element^96^, its transcriptional expression can be modified by peroxisome proliferator-activated receptors (PPARs) which have unique roles in lipid homeostasis. Indeed, PPARα and PPARγ regulate this FFA transporter in a tissue-specific manner^97^. In the liver, hepatic CD36 is increased by activating PPARα using the agonist WY14643^97,98^ and overexpressing PPARγ or inducing its activity with rosiglitazone^85,99,100^. Moreover, Zhou et al.^100^ described that CD36 is also a common target gene for other nuclear receptors: liver X receptor (LXR) and pregnane X receptor (PXR), whose activation induces hepatic steatosis in parallel to CD36 expression in mice. Interestingly, deletion of *Cd36* inhibited the effect of LXR agonists on hepatic lipid accumulation, indicating that this fatty acid translocase is crucial for hepatic steatosis onset induced by nuclear receptors. Likewise, it has been demonstrated that insulin induced hepatic CD36 expression in a PPARγ-dependent manner^89^.

In the last years, other signalling pathways have been involved in the modulation of CD36 content. For instance, AMPK activation has been reported as an inducer of CD36 expression and lipid accumulation in hepatocytes and mouse livers. This effect is mediated by activation of ERK1/2 and, subsequently, C/EBPβ, which binds to the C/EBP-response element in the *Cd36* promoter in hepatocytes^101^. Similarly, the expression of this FFA transporter is regulated by Krüppel-like factor 2 (KLF2), a transcription factor whose hepatic expression is upregulated in mice with NAFL and binds to the *Cd36* promoter inducing its activation^102^. Indeed, the overexpression through adenoviral injection of KLF2 induced marked hepatic TGs accumulation and FFA uptake, in parallel to an elevation of *Cd36* mRNA and protein levels in mice; whereas hepatocyte-specific *Klf2* deficiency improved NAFLD features in *ob/ob* mice, including the normalisation of CD36 levels^102^. Moreover, it has been demonstrated that hypoxia-inducible factors (HIFs) also modulate CD36 expression^103,104^. Particularly, HIF2α upregulated CD36 expression and function triggering lipid accumulation in hepatocytes in vitro and in vivo, thus contributing to the onset of hepatosteatosis, the earliest phase of NAFLD^105^.

Another study has described that rapamycin inhibited hepatic FFA-induced CD36 protein expression, suggesting that CD36 participates in the improvement of hepatic steatosis mediated by this inhibitor^106^. Curiously, rapamycin modulated hepatic CD36 expression at the translational, but not at the transcriptional, level since it suppressed hepatic *Cd36* translational efficiency through inhibition of the mTOR pathway, thereby resulting in a reduction of CD36 protein content^106^. Likewise, CD36 has been identified as a target of proprotein convertase subtilisin/kexin type 9 (PCSK9), which triggers its degradation through escorting the receptor to lysosomes by a mechanism involving the proteasome^107^. Consequently, mice lacking *Pcsk9* displayed hepatic steatosis features, including an elevation of CD36 protein levels although its mRNA content remained unchanged. Conversely, injection of recombinant PCSK9 to C57BL/6 mice induced the degradation of hepatic CD36^107^.

Several studies have revealed that different microRNAs (miRNAs) are capable of potentially targeting the 3′ untranslated region (UTR) of the *CD36* mRNA, so they can modulate hepatic lipid accumulation by negatively regulating this receptor. Among them, overexpression of miR-26a significantly decreased *CD36* mRNA expression and TG levels in HepG2 cells, while suppression of this miRNA displayed the opposite effects^108^. Similar results were observed modifying miR-4668 function^109^. Likewise, overexpression of miR-29a reduced protein content of CD36 in liver tissue in mice fed a HFD by targeting *Cd36* transcripts and the subsequent lipid accumulation^110^. Interestingly, miR-20b also inhibited CD36 expression and TG accumulation in FFA-treated HepG2 cella^111^. This miRNA appears to be a key component in the regulation of CD36 expression mediated by STAT5 signalling pathway since in vivo and in vitro STAT5 deletion resulted in elevated hepatic CD36 levels and lipid accumulation partly through downregulation of miR-20b^111^.

Besides hepatocytes, other liver resident cells including KCs, HSCs and LSECs express CD36^112,113^. In this regard, a decrease of HFD-induced CD36 expression in KCs may contribute to the protection against inflammation and steatosis in the liver^92,114^. Moreover, in activated HSCs, CD36 mediated-oxLDL uptake stimulates extracellular matrix synthesis. Indeed, the blockade of this receptor with a specific antibody reversed this pro-fibrogenic effect^115^.

### Role of CD36 in NAFLD

**Fig. 6:**
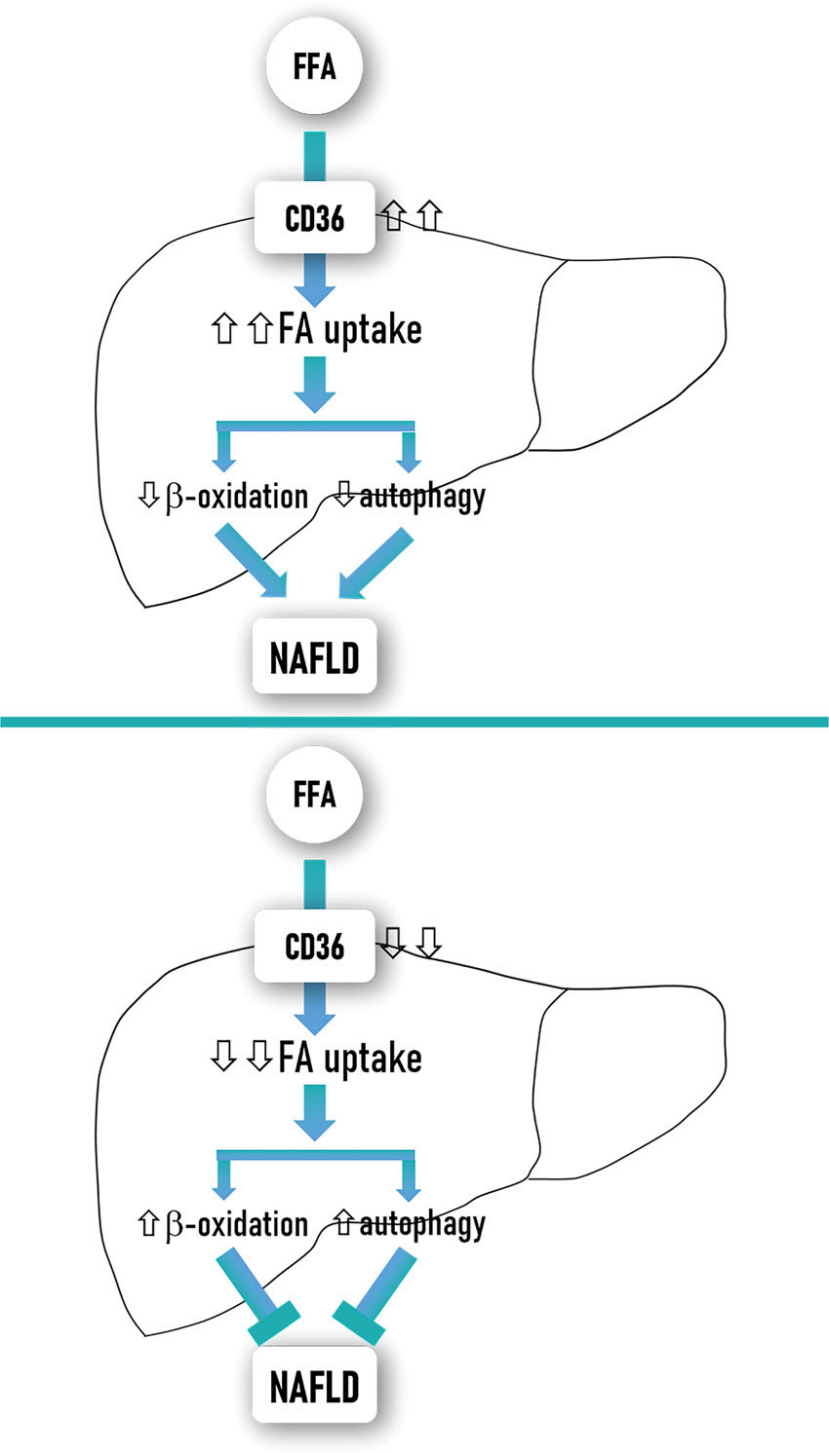
Modulation of CD36 expression in the liver directly affects NAFLD. CD36 not only acts as a FFA transporter but also regulates β-oxidation and autophagy among others lipid metabolism pathways in liver cells. Overexpression of hepatic CD36 concurs with a marked elevation of hepatic FFAs uptake and decreased β-oxidation and autophagy, thus contributing to hepatosteatosis. Conversely, downregulation of hepatic CD36 diminishes FFAs uptake and increases β-oxidation and autophagy protecting against hepatosteatosis.

The significance of CD36 in the pathogenesis of NAFLD onset has been demonstrated because modulation of its expression in the liver directly affects hepatic steatosis (Fig. 6). Overexpression of hepatic *Cd36*, achieved by using recombinant adenovirus harbouring mouse *Cd36* cDNA, was accompanied with a marked elevation in hepatic FFA uptake and TG storage in both primary hepatocytes and lean mice^88^. In contrast, mice with hepatocyte-specific *Cd36* deletion were protected against HFD-induced liver steatosis and, even, improved whole-body insulin sensitivity^116^. Furthermore, inhibition of CD36 palmitoylation decreased both CD36 expression on hepatocellular plasma membrane and hepatic TG content, protecting mice from HFD-induced NASH^84^. Noteworthy, *Cd36-*knockout mice were also protected against a high-carbohydrate liquid diet-induced hepatic steatosis but by a distinct mechanism regardless of hepatic fatty acid uptake and related to decreased expression of genes in the de novo lipogenesis pathway^81^. Intriguingly, Zhong et al.^117^ noticed that whole-body *Cd36* deletion did not affect hepatic FFAs uptake in mice while increased monocyte chemotactic protein-1 transcription in hepatocytes and enhanced hepatic inflammation and fibrosis^117^, pointing out that *Cd36* deficiency might contribute to NASH development by a fatty acid-independent mechanism. These somehow controversial findings indicate that further experimental research is needed to better understand the role of CD36 in regulating hepatic lipid homeostasis and its impact in the progression of hepatosteatosis to NASH.

Regarding human NAFLD pathophysiology, several clinical studies have attempted to clarify the role of CD36 in NAFLD onset and progression. Firstly, Greco et al*.*^118^ showed that hepatic *CD36* mRNA levels correlated with liver fat content in morbidly obese patients^118^. Likewise, it was observed an increase of *CD36* mRNA and protein content in livers from morbidly obese patients compared to healthy controls^119^. Interestingly, this study described that the abundance of this FFA transporter was significantly associated with the degree of apoptosis (TUNEL-positive cells) in the livers of the study patients. Moreover, several clinical studies have demonstrated that hepatic CD36 expression is higher in biopsy-proven NAFLD patients compared to subjects with histologically normal liver^94,120,121^.

Interestingly, a circulating form of CD36 known as soluble CD36 (sCD36) with a molecular weight similar to the membrane-bound form, was identified in human plasma. sCD36 has been proposed as a marker of altered tissue CD36 expression^122^ since its levels correlate with an overexpression of CD36 in tissues and/or cells involved in the physiopathology of the MS, such as adipocytes, myocytes and tissue macrophages^88,123,124^.

Several clinical studies conducted by Handberg et al.^125,126^ showed that sCD36 is significantly associated with different components of MS, such as markers of insulin resistance and atherosclerosis, positively correlating with the presence of these two pathologies. Indeed, sCD36 was tightly related with insulin resistance in plasma from obese patients with or without T2D, and in women with polycystic ovary syndrome^122,127,128^.

Given the close association between insulin resistance and NAFLD, it has been expected that sCD36 concentration may be increased in the plasma of these patients. In this regard, it was described that circulating sCD36 was associated with markers of liver injury, such as ALT, AST and GGT, in insulin-resistant subjects with altered glucose tolerance or T2D, but not in subjects with normal glucose homeostasis^129^. However, a cross-sectional clinical study in healthy population found that plasma sCD36 correlated with the presence of fatty liver estimated by clinical and analytical algorithms, such as fatty liver index^130^. Other clinical study has demonstrated that patients with chronic hepatitis due to HCV who showed associated steatosis had significantly higher plasma concentrations of sCD36 than those HCV-patients without steatosis^131^. Taken together these studies suggested that sCD36 could reflect hepatic steatosis, but did not demonstrate whether plasma sCD36 concentration correlates with the amount of intrahepatic fat as well as with the pattern of CD36 expression in the liver. In this connection, a previous study from our laboratory described that serum level of sCD36 increased in NAFLD patients diagnosed by liver biopsy compared to subjects with histologically normal liver, and correlated with the histological grade of steatosis, reaching the highest circulating sCD36 detected in advanced steatosis patients (grades 2 and 3)^7^. Moreover, this study demonstrated for the first time the presence of a significant correlation between circulating sCD36 and the index of hepatic expression of CD36 in NAFLD patients, suggesting that the increase in serum sCD36 values is largely due to the overexpression of CD36 observed in hepatocytes of these patients, supporting the hypothesis that the level of CD36 expression in the liver may contribute significantly to the circulating pool of sCD36^7^. Similarly, another study described that sCD36 levels were associated with the level of intrahepatic lipids (measured by magnetic resonance spectroscopy) in NAFLD patients, and close-to-significant correlated with hepatic *CD36* mRNA expression in the available biopsies from these patients^132^. Given all these data, sCD36 could be proposed as a potential biomarker of steatosis severity, and may represent a promising tool for future studies on the epidemiology, non-invasive diagnosis, treatment outcome, and prognosis of NAFLD.

### Cellular redistribution of CD36: translocation from intracellular stores to the plasma membrane

It is well-established that the function of CD36 as a FFA transporter largely depends on its localisation on the plasma membrane^133^. The first convincing evidence that increased expression of CD36 in the plasma membrane was accompanied by increased uptake and internalisation of FFAs into the cell was obtained by Bonen et al.^134^. These authors found that CD36 was expressed at both plasma membrane and the cytoplasm of resting muscle cells and, after muscle contraction induced by short electrical stimuli, there was a marked decrease in intracellular CD36 content and a significant increase in the amount of CD36 in plasma membrane. Since de novo CD36 synthesis does not occur in such a short period of time, it was concluded that the increased uptake of FFAs after muscle contraction was due to the translocation of CD36 from the cytoplasm to the plasma membrane of the muscle cells^134^. Due to this characteristic, CD36 is also known as a fatty acid translocase.

Translocation of CD36 to the plasma membrane has also been described in cardiac muscle cells as well as platelets and pneumocytes^133,135,136^. Regarding liver cells, prolonged exposure to FFAs increased not only total expression of CD36, but also triggered its translocation to the plasma membranes in rat hepatocytes^93^. Moreover, it was described that hepatocytes from obese Zucker rats, which are characterised by insulin resistance and hepatic steatosis, expressed higher amounts of CD36 in plasma membrane than in cytoplasm^137^. An interesting fact from this study was that the subcellular distribution of CD36 in the membrane only persisted when these hepatocytes were cultured with high doses of insulin, suggesting that the chronic hyperinsulinemia characteristic of obese Zucker rats could explain the translocation of CD36 to the hepatocellular membrane and, therefore, contribute to hepatic steatosis. Interestingly, a positive significant correlation between hepatic CD36 expression and plasma insulin concentrations, in parallel with a predominant localisation of CD36 at the plasma membrane, was observed in patients with NAFLD and HCV-induced steatosis^120^. In particular, in histologically normal livers, CD36 is weakly detected in the cytoplasm of hepatocytes whereas this FFA transporter is markedly expressed at the plasma membrane in addition to the cytoplasm of numerous hepatocytes in NAFLD patients^84,120^. Curiously, it has also been reported that CD36 membrane expression increased during aging in livers from both mice and humans, suggesting that it may play a key role in the development of age-associated NAFLD^121^. Since palmitoylation facilitates translocation from an intracellular pool to the plasma membrane of many proteins, a study exploring functional consequences of palmitoylation of CD36 in the liver revealed that inhibition of CD36 palmitoylation in HFD-fed mice decreased its localisation on hepatocellular plasma membrane and impaired its function as FFA transporter^84^.

### Concluding remarks and future perspectives

In the past decade, an extensive body of evidence has significantly improved understanding of the complex cellular and molecular mechanisms involved in NAFLD pathogenesis. It is now established that lipotoxic liver injury is one of the key events in NAFLD pathophysiology.

The excessive FFA influx to hepatocytes is the earliest event triggering lipotoxicity and currently it is well known that the fatty acid translocase CD36 plays a key role increasing FFA uptake and its utilisation not only in hepatocytes, but also in KCs and HSCs. There is convincing experimental evidence by studying in vitro systems and animal models of NAFLD that CD36 drives hepatosteatosis onset and might contribute to its progression to NASH. Clinical studies have reinforced the significance of CD36 in NAFLD pathogenesis in humans as hepatic CD36 content is markedly increased in NAFLD patients and, interestingly, circulating levels of a soluble form of CD36 are abnormally elevated in those patients and positively correlate with the histological grade of hepatic steatosis^138^.

While it is becoming increasingly clearer that a potential relationship between hepatic lipotoxicity and fatty acid translocase CD36 expression and function in liver cells exists, a number of questions on its impact in NAFLD progression will need to be answered in future studies. For instance, what are the molecular mechanisms involved in liver-specific regulation of CD36 expression or function, and how does this impact on hepatic lipotoxicity thus influencing progression from hepatosteatosis to NASH? Shedding light on these important topics will improve our understanding of the molecular mechanisms regulating fatty acid translocase CD36 expression and function in liver cells helping to design novel therapies that inhibit or attenuate hepatic lipotoxicity and its deleterious consequences during NAFLD.
